# Impact of Food Matrices on Digestibility of Allergens and Poorly Allergenic Homologs

**DOI:** 10.3389/falgy.2022.909410

**Published:** 2022-05-31

**Authors:** J. H. Akkerdaas, A. Cianferoni, E. Islamovic, J. Kough, G. S. Ladics, S. McClain, L. K. Poulsen, A. Silvanovich, L. Pereira Mouriès, R. van Ree

**Affiliations:** ^1^Department of Experimental Immunology, Amsterdam University Medical Centers, Amsterdam, Netherlands; ^2^Children's Hospital of Philadelphia, Philadelphia, PA, United States; ^3^BASF Corporation, Morrisville, NC, United States; ^4^US EPA, Washington, DC, United States; ^5^Dupont Nutrition and Biosciences, IFF, Wilmington, DE, United States; ^6^Syngenta Crop Protection, LLC, Greensboro, NC, United States; ^7^Copenhagen University Hospital at Gentofte, Copenhagen, Denmark; ^8^Bayer U.S. Crop Science, Chesterfield, MO, United States; ^9^Health & Environmental Sciences Institute (HESI), Washington, DC, United States; ^10^Department of Otorhinolaryngology, Amsterdam University Medical Centers, Amsterdam, Netherlands

**Keywords:** food matrix, protease resistance, allergenicity, allergen exposure, risk assessment

## Abstract

**Background:**

Protease resistance is considered a risk factor for allergenicity of proteins, although the correlation is low. It is nonetheless a part of the weight-of-evidence approach, proposed by Codex, for assessing the allergenicity risk of novel food proteins. Susceptibility of proteins to pepsin is commonly tested with purified protein in solution.

**Objective:**

Food proteins are rarely consumed in purified form. Our aim was to evaluate the impact of experimental and endogenous food matrices on protease susceptibility of homologous protein pairs with different degrees of allergenicity.

**Methods:**

Porcine and shrimp tropomyosin (ST) were subjected to sequential exposure to amylase, pepsin, and pancreatin in their respective endogenous matrix (pork tenderloin/boiled shrimp) and in three different experimental matrices (dessert mousse [DM], soy milk [SM], and chocolate bar [CB]). Digestion was monitored by immunoblotting using tropomyosin-specific antibodies. Recombinant peach and strawberry lipid transfer protein were biotinylated, spiked into both peach and strawberry fruit pulp, and subjected to the same sequential digestion protocol. Digestion was monitored by immunoblotting using streptavidin for detection.

**Results:**

Chocolate bar, and to a lesser extent SM, had a clear protective effect against pepsin digestion of porcine tropomyosin (PT) and to a lesser extent of ST. Increased resistance was associated with increased protein content. Spiking experiments with bovine serum albumin (BSA) confirmed the protective effect of a protein-rich matrix. The two tropomyosins were both highly resistant to pepsin in their protein-rich and lean native food matrix. Pancreatin digestion remained rapid and complete, independent of the matrix. The fat-rich environment did not transfer protection against pepsin digestion. Spiking of recombinant peach and strawberry lipid transfer proteins into peach and strawberry pulp did not reveal any differential protective effect that could explain differences in allergenicity of both fruits.

**Conclusions:**

Protein-rich food matrices delay pepsin digestion by saturating the protease. This effect is most apparent for proteins that are highly pepsin susceptible in solution. The inclusion of food matrices does not help in understanding why some proteins are strong primary sensitizers while homologs are very poor allergens. Although for induction of symptoms in food allergic patients (elicitation), a protein-rich food matrix that may contribute to increased risk, our results indicate that the inclusion of food matrices in the weight-of-evidence approach for estimating the potential risks of novel proteins to become allergens (sensitization), is most likely of very limited value.

## Introduction

Resistance to gastro-intestinal proteolysis is generally considered to be one of the factors deciding on whether a food protein will likely behave as a food allergen or not. In line with that, pepsin digestion assays are an integral part of the weight-of-evidence approach for allergenicity risk assessment of genetically modified (GM) crops (1). Although a correlation between resistance to pepsin digestion and allergenic potential has been proposed (2), the correlation is low (3–5). We have recently reported that resistance to pepsin and pancreatin is in fact poor predictors of allergenicity (6). In that study, pairs of established allergens and weakly or non-allergenic homologs were subjected to sequential digestion with amylase, pepsin, and pancreatin. For pepsin digestion, different pH and pepsin-to-protein ratios were tested, to account for the variety of conditions occurring in real life. Although under optimal conditions (i.e., low pH/high pepsin), in particular Ara h 2, and to a lesser extent Pru p 3 and Pen a 1, were more resistant to pepsin than their poorly allergenic relatives, the established fish allergen parvalbumin was very sensitive to pepsin digestion. Under less favorable conditions (i.e., higher pH/lower pepsin), all proteins were similarly resistant. On the other hand, all tested allergenic and non-allergenic proteins proved to be very sensitive to pancreatin digestion, except fish parvalbumin that was highly resistant. Overall, the picture emerging is that assays for gastro-intestinal digestion cannot reliably distinguish allergens from non-allergens, certainly not under sub-optimal conditions for pepsin that frequently occur in real life. This implicates that protease resistance is unlikely to be a useful outcome to predict the risk that a novel protein will become an allergen, i.e., it is likely to induce de-novo sensitization. Such a poor predictive performance is better in line with the concept that sensitization to food allergens can occur *via* the skin, where gastro-intestinal proteolytic enzymes do not play a role (7).

This does, however, not mean that pepsin and pancreatin resistance are irrelevant in the weight-of-evidence approach for allergenicity risk assessment. Elicitation of (potentially severe) systemic symptoms in already sensitized subjects is dependent on the degree to which sufficiently intact allergen reaches the gut immune system. The likelihood of sufficient allergen reaching the gut immune system is dependent on a combination of the abundance of the allergen in a (composite) food and its inherent stability to pepsin and pancreatin (8), but also by the type of food processing (9). Good examples of the latter are differences observed in allergenicity between raw and baked milk and egg (10) and between roasted and boiled peanuts (11). Overall, resistant and abundant proteins, such as 2S albumins in tree nuts, legumes, and seeds, are more likely to induce systemic symptoms than very labile proteins, such as pathogenesis-related class 10 (PR-10) proteins, present at mostly very low levels in a broad spectrum of plant foods, such as fruits, vegetables, tree nuts, and legumes (12–15). To what extent an allergen gets the chance to elicit systemic symptoms will not only depend on their inherent resistance to proteolysis, their abundance in a serving, and the type of food processing but may also be affected by the context in which the protein is consumed. Co-factors that have been described to increase the risk of (severe) allergy symptoms range from the use of alcohol, the use of non-steroidal anti-inflammatory drugs, the use of antacids and exercise (16). Other factors that have been implicated to have a potential impact on the allergenicity of foods are different processing methods (17–19) and (the composition of) food matrices (20). In the case of composite processed foods, unraveling this can be a very complicated multifactorial puzzle. To date, not many reports have addressed the role of food matrices in protease digestion. Schulten et al. reported delayed digestion of food allergens in the presence of protein-rich food (hazelnut) extract as a surrogate for food matrix (21). Another study showed that hydrogel-forming pectin from fruits could inhibit pepsin digestion (22). Prodic et al. performed digestion protocols on solid milled peanut concluding that major allergens remain intact, but they did not compare susceptibility with peanut allergens in solution (23). Two different food matrices, a protein-poor chocolate dessert and a baked cookie, were reported to have a limited impact on the digestion of roasted peanut flour (24). Torcello-Gomez et al. investigated matrix effects in a very extensive comparison of gastric and intestinal digestion of purified milk and egg allergens and the respective whole foods, using three protocols representing the state of the digestive tract at infant, early, and late adult age (25). In addition, recently, Mattar et al. studied digestibility and bio-accessibility of major egg and peanut allergens, when whole egg and peanut were digested in a baked muffin matrix (26). The broad variety in experimental designs of these studies, and the complexity that composition and degree of processing of different food matrices add to that, prevents from drawing simple conclusions about the impact of food matrix.

To further characterize factors associated with food matrices that may contribute to altered resistance of allergens to proteolysis, we here compared the stability of shrimp and porcine tropomyosin (PT), in their natural food matrix and spiked into three different matrices with different compositions with respect to protein, fat, and carbohydrate. In addition, we have compared proteolysis by (cross-) spiking biotinylated recombinant lipid transfer proteins from a highly allergenic (peach) and a weakly allergenic (strawberry) fruit in the pulp of both fruits. The aim of our study was 2-fold: (1) to investigate whether food matrices can contribute to differences in allergenicity that may go undetected when subjecting purified proteins in solution to digestion protocols and (2) to elucidate which category of food components, i.e., protein, fat, and/or carbohydrate, has an impact on the resistance of allergens to proteolysis.

## Materials and Methods

### Reagents

All enzymes (amylase, pepsin, and pancreatin) and PT were purchased from Sigma Aldrich (Saint Louis, MO, USA). Shrimp tropomyosin (ST) was obtained from Indoor Biotechnologies (Charlottesville, VA, USA). Soy milk (SM; Alpro, Danone, Paris, France) and chocolate bars (CBs) with 85% cacao (CB; Lindt, Oloron-Sainte-Marie, France) were purchased at a local supermarket. The dessert mousse (DM) (27) was kindly provided by Reacta Healthcare (Deeside, UK). The protein, fat, and carbohydrate contents of the three test matrices are listed in Table 1.

**Table 1:** Composition of food matrices.

		**Matrix**	

**Composition**	**DM**	**SM**	**CB**
Protein	1.7%	3.0%	12.5%
Fat	10.0	1.8%	46.0%
Carbohydrate	11.6%	2.5%	19.0%
Water	76.7%	92.7%	22.5%

*The table provides percentages of the total weight with respect to protein, fat, carbohydrate, and water for the three food matrices used in the digestion experiments: DM, dessert mousse; SM, soy milk; CB, chocolate bar. Increasing darkness of each color reflects increasing percentage*.

### Sequential Digestion Protocol

The sequential digestion protocol used in this study was essentially identical to the one reported by Minekus et al. (28), with a salivary phase in simulated salivary fluid (SSF) at pH7, a gastric phase in simulated gastric fluid (SGF) at pH3, and an intestinal phase in simulated intestinal fluid (SIF) at pH7. The composition of SSF, SGF, and SIF was identical to those described by Minekus et al.

In brief, digestion was performed as follows: shrimp and PT (125 μl/1.2 mg/ml) were incubated under continuous rotation (750 rpm) at 37°C with 125 μl matrix material (SM, CB, and DM), 125 μl of amylase solution (2 mg/ml in SSF), 75 μl SSF, 12.5 μl 30 mM CaCl_2_, and 37.5 μl H_2_O, adding up to a total volume of 500 μl. After 2 min, a 15-μl sample was drawn from the amylase digest. Amylase digestion was immediately stopped by the addition of 85 μl sodium dodecyl sulfate-polyacrylamide gel electrophoresis (SDS-PAGE) sample buffer (Thermo Fisher Scientific, Carlsbad, CA, USA).

Immediately thereafter, 375 μl of the amylase digest was added to 7.5 μl 1M HCl, 70.4 μl pepsin (21,300 U/ml in SGF), 270.5 μl SGF, 2 μl 30 mM CaCl_2_, and 24.6 μl H_2_O, adding up to a total volume of 750 μl (pH 2.5–3.0/pepsin to tropomyosin ratio >13.3 U/μg). At *t* = 5, 10, 60, and 120 min, 30 μl samples were drawn, and digestion was stopped by the addition of 70 μl of SDS-PAGE sample buffer.

Finally, 375 μl of the remaining SGF digest was incubated with 31.25 μl pancreatin (24 mg/ml in SIF), 268.75 μl SIF, 46.8 μl bile salt buffer (30 mM sodium taurocholate hydrate, 30 mM sodium glycodeoxycholate [both Sigma] in 50 mM KH_2_PO_4_/K_2_HPO_4_ phosphate buffer, pH7.5), 7.5 μl CaCl_2_, 2.8 μl 1M NaOH, and 18 μl H_2_O. At *t* = 10, 60, and 120 min, 60 μl samples were drawn and digestion was stopped by the addition of 40 μl SDS-PAGE sample buffer.

Samples were separated on 4–12% SDS-PAGE Bis-Tris gels (Invitrogen) and subsequently transferred to nitrocellulose for immunoblot analyses with either a monoclonal antibody against chicken tropomyosin, cross-reactive with PT (Thermo Scientific, Rockland, IL, USA) or monoclonal antibody 1A6 against house dust mite tropomyosin, cross-reactive with ST (29). The binding of both monoclonals was detected using anti-mouse IRDye800 using the Odyssey Imaging System (LI-COR Biosciences, Miami, FL, USA).

### Spiking Experiments With Protein and Fat

To elucidate which matrix components are most likely explaining different degrees of protection was observed in the presence of the three matrices, a spiking approach with protein, and fat was applied to the matrix lowest in protein (DM) and to the one lowest in fat (SM), respectively. To investigate a potential protective role of protein content, PT was digested using the sequential digestion protocol in DM (1.75% protein) and in the same matrix spiked with 1.25% bovine serum albumin (BSA; Roche Diagnostics GmbH, Mannheim, Germany) to mimic the protein content of SM (3% protein) or with 10.75% BSA to mimic the protein content of the CB matrix (12.5% protein). To investigate a potential protective role of fat content, PT was digested in SM (1.8% fat) and in the same matrix spiked with 8.2% fat (Choc chick raw organic cacao butter/99.8% fat; Holland & Barrett, Warwickshire, UK) to mimic the fat content of DM (10% fat) or with 44.2% fat to mimic the fat content of the CB (46% fat).

### Digestion of Tropomyosins and Lipid Transfer Proteins (LTPs) in Their Natural Matrices

To investigate the impact of natural matrices on the digestion of tropomyosins, 125 mg of shrimp grounded meat (2% fat/15–20% protein) and 125 g of grounded pork tenderloin (2% fat/22–23% protein) were digested in SGF and after 2 h transferred to SIF and subsequently digested for 2 h. Samples were taken at 5, 10, 60, and 120 min during the gastric phase and at 10, 60, and 120 min during the intestinal phase. Digestion was monitored by immunoblot as described above.

To compare the impact of natural fruit matrices on the digestibility of lipid transfer proteins from peach (Pru p 3) and strawberry (Fra a 3), a different approach was taken. Recombinant versions of both allergens (6) were biotinylated with EZ-Link sulfo-NHS-LC-biotin (Thermo Scientific) according to the manufacturer's instructions at a molar biotin over protein ratio of 10. Subsequently, 250 mg of fruit pulp from peach and from strawberry were both blended together with 10 μg rFra 3^biot^ or 10 μg rPru p 3^biot^, resulting in two homologous mixes and two heterologous mixes. All four mixes were subjected to the same sequential digestion protocol as described before. Detection on immunoblot was carried out with streptavidin-IRDye800 using the Odyssey Imaging System (LI-COR).

## Results

**Figure 1:**
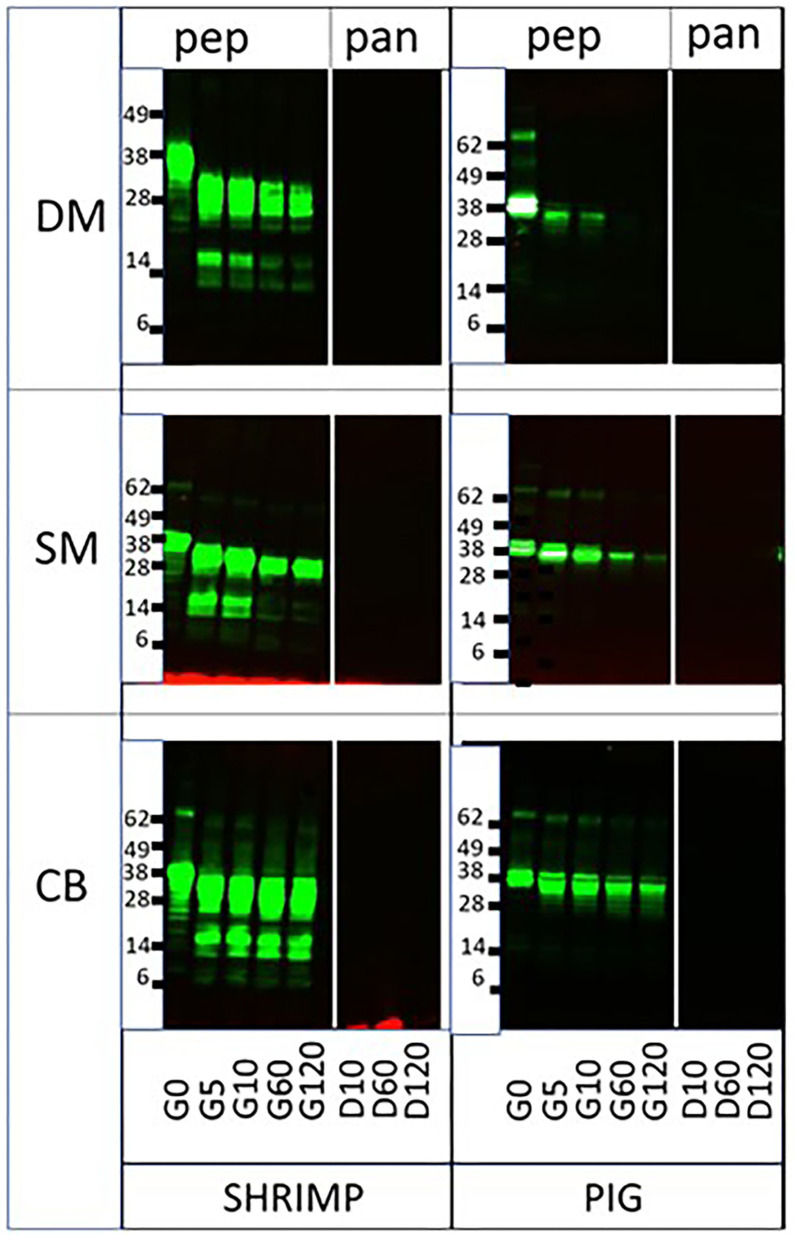
Sequential pepsin-pancreatin digestion of shrimp and porcine tropomyosin in three matrices. DM, dessert mousse; SM, soy milk; CB, chocolate bar, pep, pepsin; pan, pancreatin. Samples were taken at the start of pepsin digestion (G [gastric] 0) and at 5 (G5), 10 (G10), 60 (G60), and 120 (G120) min, and at 10 (D [duodenal] 10), 60 (D60), and 120 (D120) min of subsequent duodenal digestion. Samples were analyzed by immunoblot with respective tropomyosin-specific antibodies. Molecular weight markers are indicated on the side in kDa.

To evaluate the impact of different food matrices on protease resistance of an allergen and homologous non-allergen pair, we subjected ST and PT to sequential salivary, gastric, and intestinal digestion in DM, SM, and CB. These matrices were chosen based on their divergence in protein, fat, and carbohydrate content (Table 1). Earlier, we had demonstrated that ST was significantly more resistant to pepsin than its porcine homolog. While the latter was fully digested at 5 min at suboptimal higher pH4 and lower pepsin to protein ratios, ST was fully resistant up to at least 1 h under those conditions (6). Resistance to the digestion of ST did not diverge very much between the three matrices, using pepsin-favorable conditions (pH 2.5–3.0/pepsin to tropomyosin ratio of 13.3; Figure 1). Perhaps only in CB, a prolonged presence at 60 and 120 min of lower molecular weight (MW) breakdown peptides was observed during pepsin digestion, when compared to the digestion in SM and DM. In contrast, resistance to pepsin of PT was clearly increased as compared to digestion in solution (6) and differed between the three matrices, i.e., lowest in DM and highest in CB. In CB, the PT band was still clearly detected at 2 h, in SM at 1 h and weakly at 2 h, and finally in DM still at 10 min. This order of increasing resistance is in parallel with increasing protein content (Table 1). This suggests that a protein-rich environment protects the test protein against pepsin digestion.

**Figure 2:**
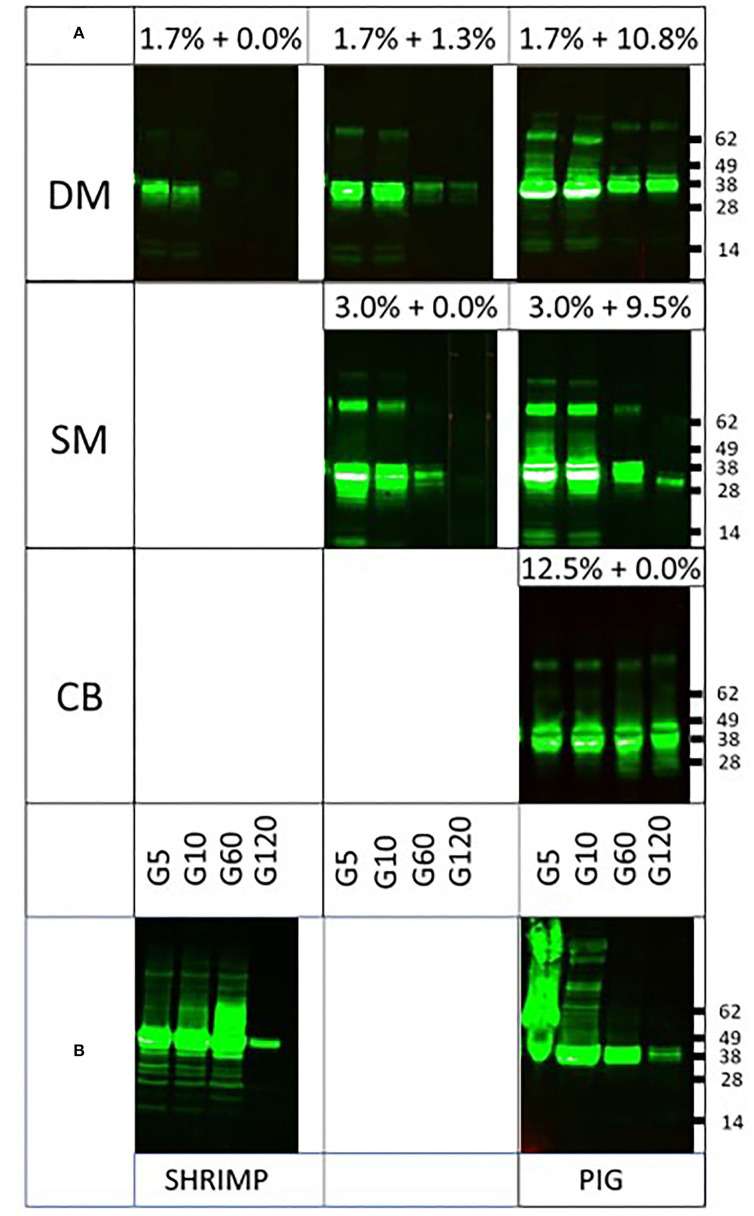
Impact of extra protein from spiking or natural matrix on pepsin digestion of tropomyosins. DM, dessert mousse; SM, soy milk; CB, chocolate bar. Samples were taken at 5 (G5), 10 (G10), 60 (G60), and 120 (G120) min of pepsin digestion. Samples were analyzed by immunoblot with respective tropomyosin-specific antibodies. Molecular weight markers are indicated on the side in kDa. **(A)** Impact on pepsin digestion of tropomyosins of the addition of BSA to DM to the percentage of protein found in SM and CB, respectively, and of addition of BSA to SM to the percentage of protein found in CB. **(B)** Impact on pepsin digestion of tropomyosins by their respective endogenous protein-rich matrices.

**Figure 3:**
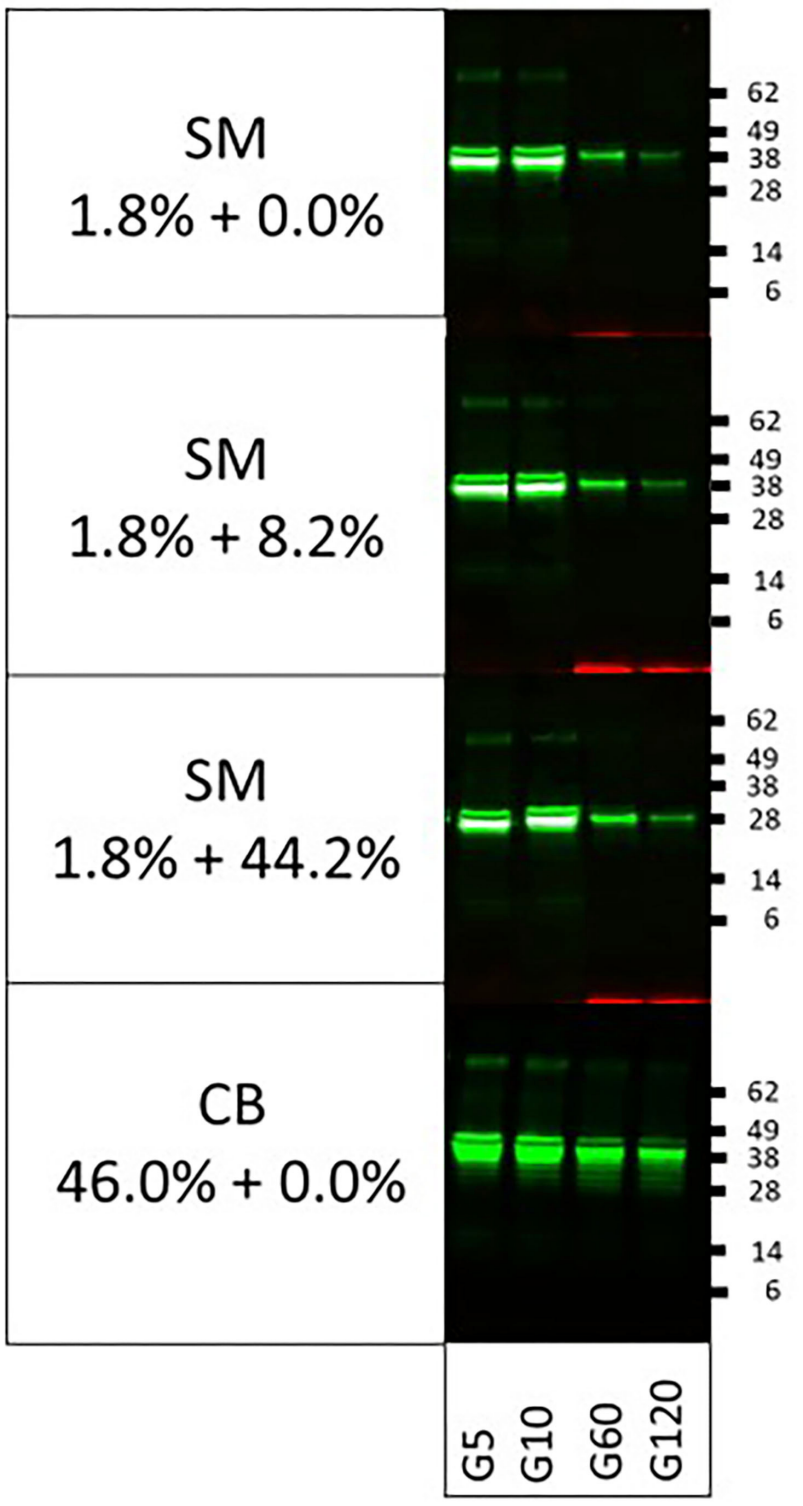
Impact of the addition of extra fat to soy milk on pepsin digestion of porcine tropomyosin. DM, dessert mousse; SM, soy milk; CB, chocolate bar. Samples were taken at 5 (G5), 10 (G10), 60 (G60), and 120 (G120) min. Samples were analyzed by immunoblot with respective tropomyosin-specific antibodies. Molecular weight markers are indicated on the side in kDa. Cocoa butter was added to SM to reach the percentage of fat reported for the DM and CB matrices, respectively.

To investigate whether this is indeed the case and whether fat content may additionally play a protective role, being highest in CB as well, we performed spiking experiments using BSA and cocoa butter as sources of protein and fat, respectively. When DM (1.7% protein) was replenished with BSA to levels of protein present in SM (3.0%) or CB (12.5%), clearly resistance of tropomyosin increased to levels similar to those observed in SM and CB, respectively (Figure 2A). A similar observation was made upon spiking of SM to the protein level of CB (Figure 2A). To further provide support for a protective role of protein, we next performed digestion of both tropomyosins in their natural matrix. Both proteins were still clearly detected at 2 h of pepsin digestion (Figure 2B). To investigate whether fat added any further protection against digestion, PT was subjected to pepsin in SM (1.8% fat) spiked with cocoa butter to levels of fat present in DM (10%) and CB (46%), respectively. Between un-spiked and both spiked SM matrices, no differences were observed in protease resistance (Figure 3). The addition of fat to a level observed in CB did not result in similar resistance as observed in CB.

**Figure 4:**
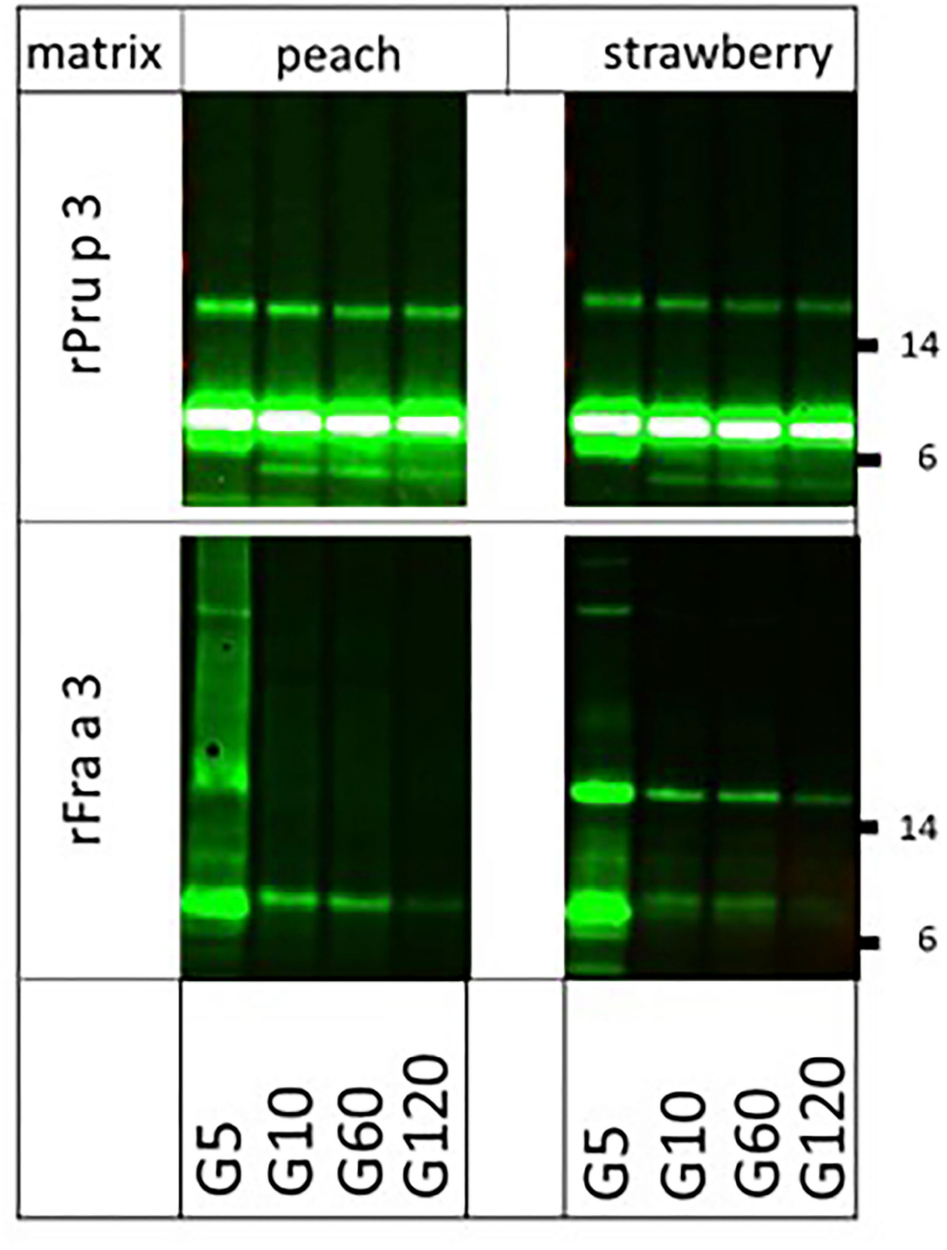
Impact of natural fruit matrices on pepsin susceptibility of homologous fruit lipid transfer protein (LTPs) Biotinylated recombinant LTPs from peach (rPru p 3) and strawberry (rFra a 3) were spiked into their endogenous fruit matrix and into each other's fruit matrix. Samples were taken at 5 (G5), 10 (G10), 60 (G60), and 120 (G120) min of pepsin digestion. Immunoblot analysis was carried out with labeled streptavidin. Molecular weight markers are indicated on the side in kDa.

**Figure 5:**
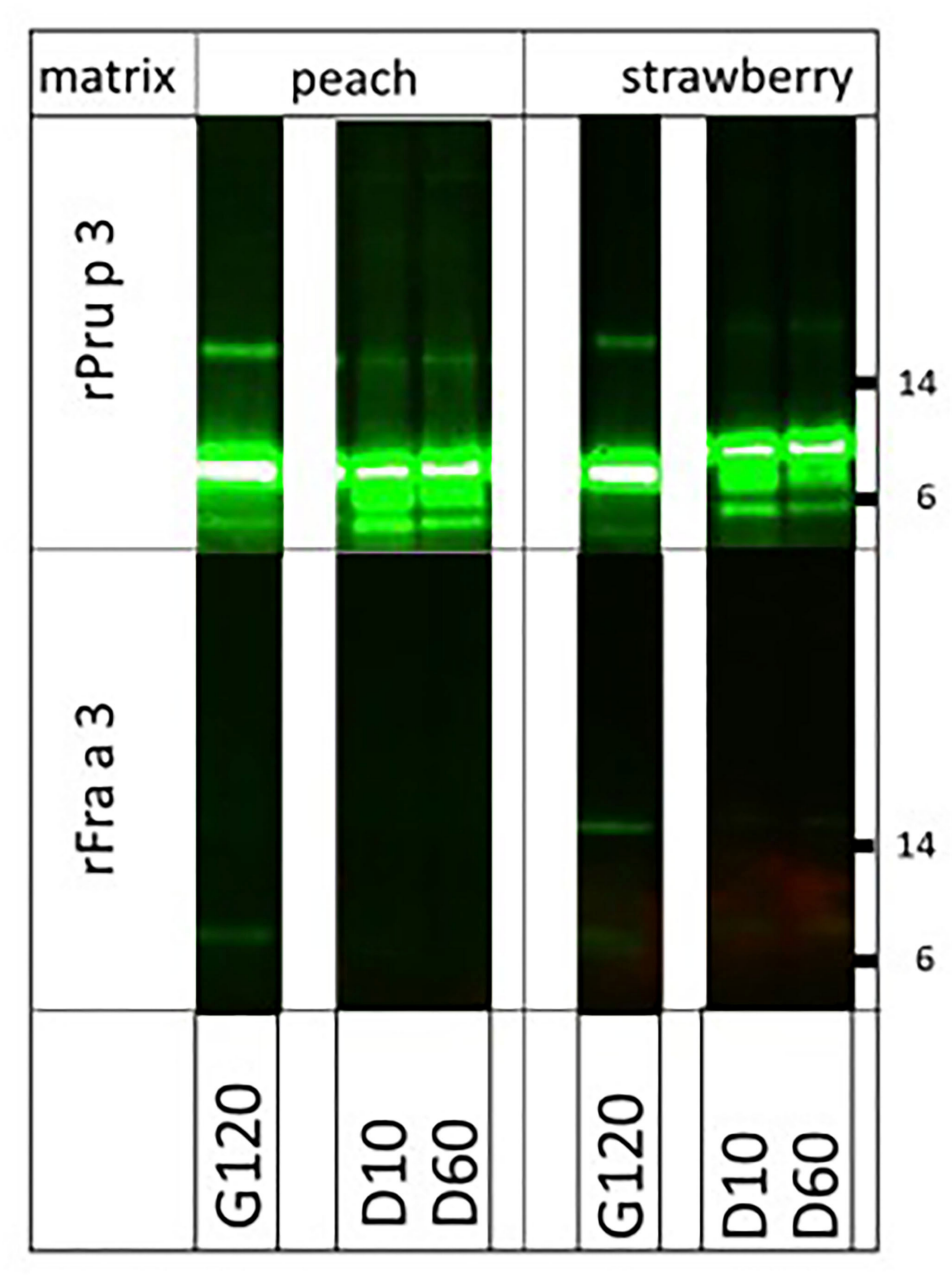
Impact of natural fruit matrices on pancreatin susceptibility of homologous fruit lipid transfer protein (LTPs). Biotinylated recombinant LTPs from peach (rPru p 3) and strawberry (rFra a 3) were spiked into their endogenous fruit matrix and into each other's fruit matrix. Samples were taken during pancreatin digestion (after 2 h pepsin digestion) at G120 (=D0), and at 10 (G10), and 60 (G60) min of pancreatin digestion. Immunoblot analysis was carried out with labeled streptavidin. Molecular weight markers are indicated on the side in kDa.

Finally, we were interested in knowing whether the very clear difference in allergenicity of two structurally very similar molecules (67% sequence identity), the highly allergenic peach LTP (Pru p 3) and the poorly allergenic strawberry LTP (Fra a 3), could (partly) be explained by the impact of their natural fruit matrices. To that end, we spiked both endogenous matrices (fruit pulps) with equal amounts of biotinylated rPru p 3 and rFra a 3 and performed the digestion again (Figure 4). Peach LTP, both the main monomeric and the minor dimeric band, proved fully resistant to pepsin in peach pulp and strawberry pulp, as had previously been shown to be the case in solution (6). For strawberry LTP, the monomeric band was similarly susceptible to digestion in both fruit pulps, resembling the earlier reported pattern of digestion in solution (6). Surprisingly, the Fra a 3 dimer was more resistant to strawberry pulp than to the peach pulp. In solution, Pru p 3 had proven to be resistant to pancreatin as well, whereas Fra a 3 was not. In both fruit pulps, the difference between both LTPs did not change: Pru p 3 was resistant in both peach and strawberry pulp, and Fra a 3 was highly susceptible to pancreatin (Figure 5). Overall, the higher allergenicity of Pru p 3 can therefore not be explained by properties transferred by its endogenous matrix.

## Discussion

In the present study, we have addressed the potential role of food matrices in the digestibility of food proteins with different degrees of allergenicity. Allergenicity can be viewed in two ways, i.e., as the likelihood that a protein will induce *de novo* sensitization and as the likelihood that a protein will induce symptoms in already sensitized subjects. As we have earlier shown, *in vitro* resistance to gastrointestinal digestion of proteins in solution is a poor predictor to establish whether a protein will become an allergen or not (6). In the same study, we did however observe significant differences between tested proteins in resistance to pepsin and pancreatin and concluded that this bears relevance for estimating the risk that an already sensitized patient is exposed to a sufficiently large quantity of an allergen for inducing (potentially severe systemic) symptoms. Exposure to an abundant allergen in a food, in combination with a high degree of resistance to digestion, may increase the risk of experiencing a reaction in those specific populations. From that perspective, *in vitro* digestion experiments remain a relevant element for the weight-of-evidence approach to assess the risk of allergenicity of novel proteins. Here, we tried to address the potential impact of food matrices on both aspects of allergenicity.

To investigate whether food matrices may help in answering the question “*why some food proteins are capable of inducing immunoglobulin E (IgE) whereas others are less likely to do so?”*, we here focused on two pairs of proteins that we had previously subjected to extensive *in vitro* digestion assays in solution (6), i.e., in the absence of any food matrix: shrimp and PTs and peach and strawberry lipid transfer proteins. In these earlier studies, we demonstrated that ST and peach LTPs were more resistant to pepsin than their non- or weakly allergenic homologs from pig and strawberry, respectively. Although only based on these two pairs of acknowledged strong allergens (ST and peach LTP) and non- or weakly allergenic homologs (PT and strawberry LTP), we found no convincing support that the endogenous food matrices of these proteins contribute to their difference in allergenicity. When digested in their natural (protein-rich) food matrices, shrimp and PTs were similarly resistant to pepsin, staying both clearly detectable up to 2 h of pepsin exposure. This implies that the lack of allergenicity of pork meat as compared to shrimp meat for tropomyosin-sensitized patients cannot be explained by better protection against pepsin digestion provided by the shrimp matrix. On the other hand, as reported for digestion in solution, pancreatin fully digested both tropomyosins almost instantly (not shown). In addition, for both LTPs, digestion in their natural fruit matrices did not contribute to explaining why peach is so much more relevant as an allergenic food than strawberry. Peach LTP was similarly stable to pepsin in its endogenous matrix (peach pulp) as in strawberry pulp, and if anything, strawberry LTP was perhaps slightly more stable in its endogenous matrix than in peach pulp. As was shown for both LTPs in solution (6), peach LTP was resistant to pancreatin in both fruit pulps whereas strawberry LTP was readily digested. These results indicate that the fruit matrix does not explain the difference in allergenicity of both fruits.

Although based on only two pairs of proteins, these results indicate that the inclusion of food matrices in the weight-of-evidence approach for estimating the potential risks of novel proteins to become allergens (sensitization) is most likely of very limited value. On the other hand, our analyses have demonstrated that protein-rich matrices saturate pepsin, allowing susceptible proteins to escape digestion for a longer time, leading to an increased risk of exposure (elicitation). Even though this was particularly apparent for PT, it was to a lesser extent also observed for ST. The protective effect was clearly associated with the protein content of the three experimental and two endogenous food matrices. In spiking experiments with BSA, the protective (pepsin-saturating) role of protein could further be substantiated. While fat content has earlier been proposed to play a role in the availability of allergen (30) and threshold for induction of symptoms (31), it could not be demonstrated to transfer a protective effect. The addition of cocoa butter to low-fat SM did not alter the susceptibility of PT to pepsin.

In conclusion, differences in sensitizing potential of proteins (i.e., known clinical differences between the pairs of proteins in this study) are not explained by their physical resistance to digestion when placed in food matrices. Results from this study indicate that the assessment of the influence of food matrices does not provide additional or new evidence regarding “*de novo*” allergenic potential of a newly expressed protein (compared to the established *in vitro* protein digestibility assessment), and therefore, the inclusion of food matrices in the weight-of-evidence approach for estimating the potential risks of novel proteins to become allergens would be of very limited value. Higher experimental protein content surrounding allergens does however increase their resistance to pepsin digestion. Presumably, increased oral and gastric exposure to allergens under the high protein matrix scenario would increase the risk of eliciting symptoms in already sensitized food allergic subjects. From this perspective, assessing susceptibility to gastrointestinal digestion is of potential value for an *in vitro* approach to estimate exposure risks of known protein allergens. In combination with established allergenic properties of a protein, stability adds potential risk characterization data for certain individuals, but as an isolated biophysical property protease resistance remains unproven to add to the characterization of a protein to become a new allergen. Perhaps this is the logical consequence of food sensitization, the key developmental step in establishing the potential for symptomatic elicitation, being a largely skin-driven process where gastro-intestinal digestion plays no role.

## Data Availability Statement

The original contributions presented in the study are included in the article/supplementary material, further inquiries can be directed to the corresponding author/s.

## Author Contributions

JA: execution of all experiments, contributed to writing manuscript, reviewing, and approval manuscript. AC, JK, and LPo: reviewing and approval manuscript. EI, GL, SM, AS, and LPe: reviewing, editing, and approval manuscript. RR: design of study, writing of manuscript, and approval manuscript. All authors contributed to the article and approved the submitted version.

## Funding

This research was funded by the Protein Allergenicity, Toxins & Bioinformatics (PATB) Committee of HESI. HESI is a non-profit scientific organization that facilitates public and private partnerships in human and environmental health. The PATB Committee is committed to advancing the scientific understanding of the relevant parameters defining allergenic proteins and protein toxins and to encourage the development of reliable and accurate methodologies for characterizing the allergenic potential of novel proteins.

## Conflict of Interest

EI was employed by BASF Corporation. GL was employed by Dupont Nutrition and Biosciences. SM was employed by SAS Institute. AS was employed by Bayer U.S. Crop Science. RR was reported consultancy for HAL Allergy, Citeq, Angany, Reacta Healthcare, Mission MightyMe, AB Enzymes. The remaining authors declare that the research was conducted in the absence of any commercial or financial relationships that could be construed as a potential conflict of interest.

## Publisher's Note

All claims expressed in this article are solely those of the authors and do not necessarily represent those of their affiliated organizations, or those of the publisher, the editors and the reviewers. Any product that may be evaluated in this article, or claim that may be made by its manufacturer, is not guaranteed or endorsed by the publisher.

## Abbreviations

BSA: bovine serum albumin
CB: chocolate bar
DM: dessert mousse
LTP: lipid transfer protein
SGF: simulated gastric fluid
SIF: simulated intestinal fluid
SM: soy milk
SSF: simulated saliva fluid
ST: shrimp tropomyosin.
